# Supplementary material for: Verteporfin-Loaded Lipid Nanoparticles Improve Ovarian Cancer Photodynamic Therapy In Vitro and In Vivo
Source: Cancers (Basel). 2019 Nov 8;11(11):1760. doi: 10.3390/cancers11111760 (PMC6896159; doi:10.3390/cancers11111760)
Supplement: Supplementary file 1 [file cancers-11-01760-s001.zip › cancers-607911-supplementary-final.docx]

Supplementary Materials

Verteporfin-Loaded Lipid Nanoparticles Improve Ovarian Cancer Photodynamic Therapy In Vitro and In Vivo

Thierry Michy ^1,2^, Thibault Massias ^1^, Claire Bernard ^1,2^, Laetitia Vanwonterghem ^1^, Maxime Henry ^1^, Mélanie Guidetti ^1^, Guy Royal ^3^, Jean-Luc Coll ^1^, Isabelle Texier ^4^, Véronique Josserand ^1,†,^*, and Amandine Hurbin ^1,†,^*

Supplementary Figures


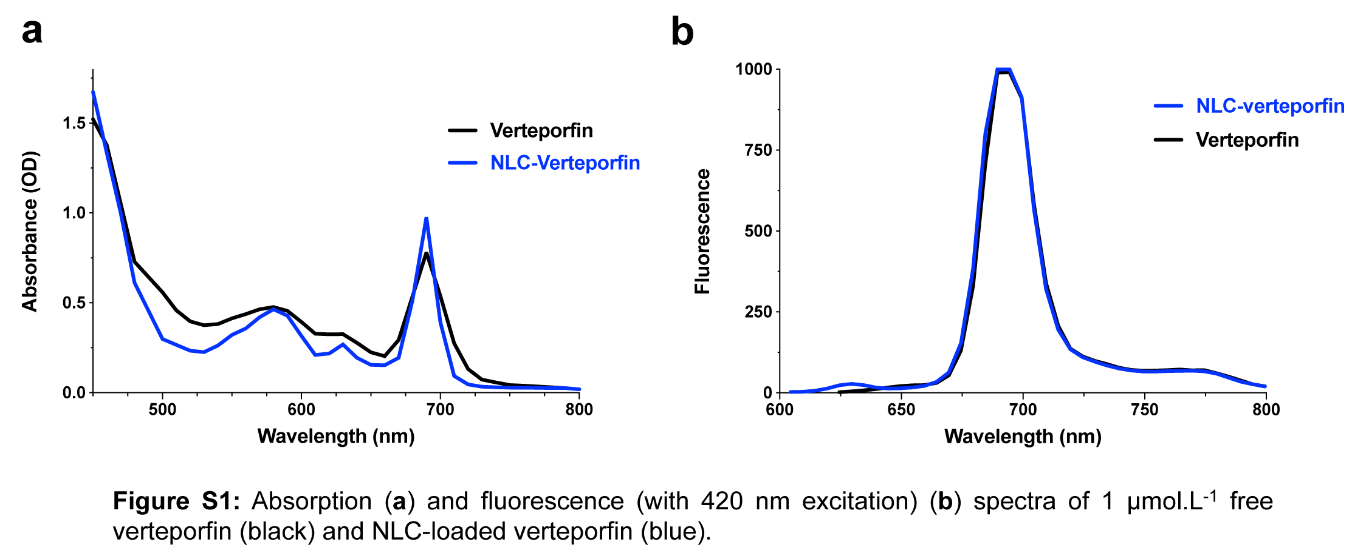


**Figure S1.** Absorption (**a**) and fluorescence (with 420 nm excitation) (**b**) spectra of 1 µmol.L^−1^ free verteporfin (black) and NLC-loaded verteporfin (blue).


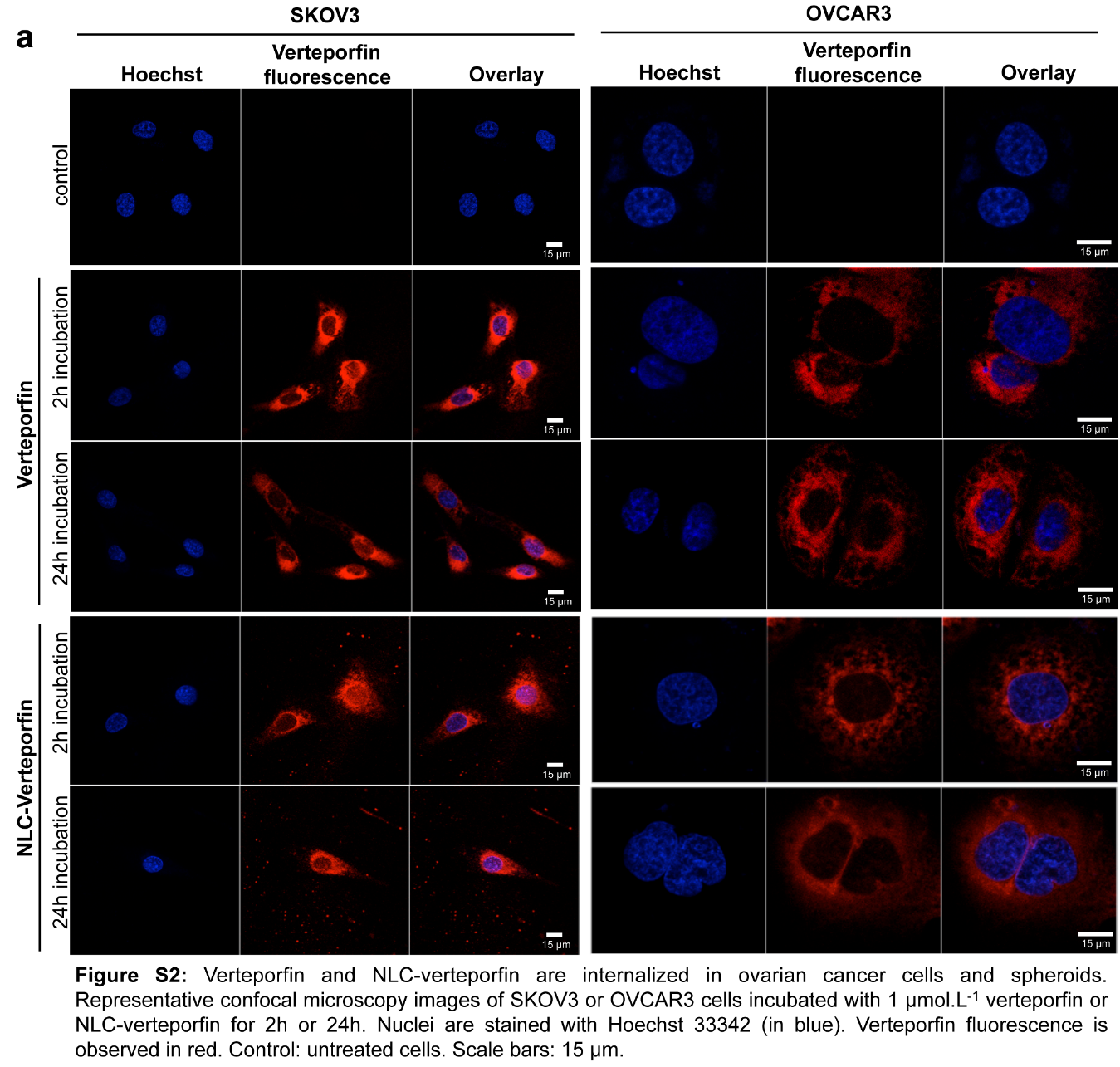


**Figure S2.** Verteporfin and NLC-verteporfin are internalized in ovarian cancer cells and spheroids. Representative confocal microscopy images of SKOV3 or OVCAR3 cells incubated with 1 µmol.L^−1^ verteporfin or NLC-verteporfin for 2 h or 24 h. Nuclei are stained with Hoechst 33342 (in blue). Verteporfin fluorescence is observed in red. Control: untreated cells. Scale bars: 15 µm.


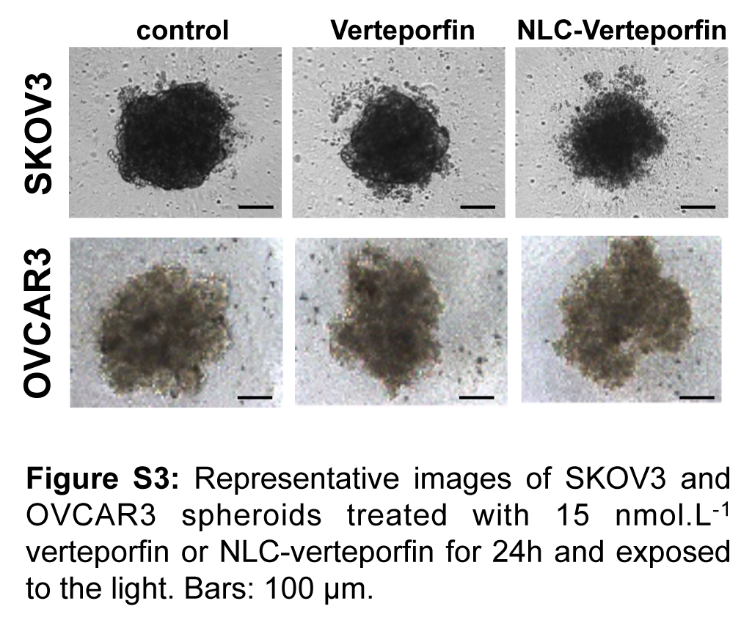


**Figure S3.** Representative images of SKOV3 and OVCAR3 spheroids treated with 15 nmol.L^−1^ verteporfin or NLC-verteporfin for 24 h and exposed to the light. Bars: 100 µm.


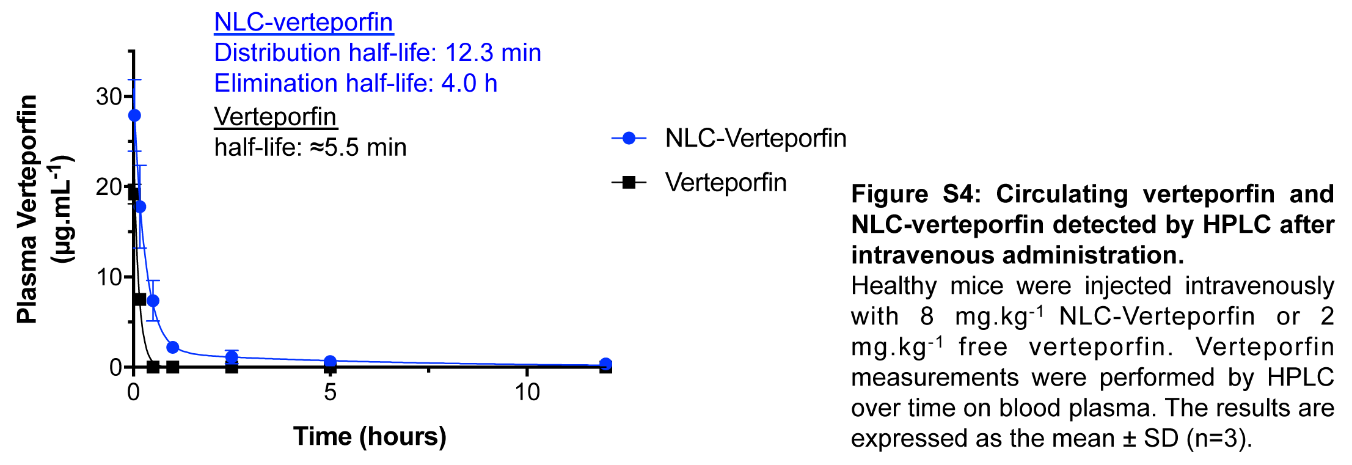


**Figure S4.** Circulating verteporfin and NLC-verteporfin detected by HPLC after intravenous administration. Healthy mice were injected intravenously with 8 mg.kg^−1^ NLC-Verteporfin or 2 mg.kg^−1^ free verteporfin. Verteporfin measurements were performed by HPLC over time on blood plasma. The results are expressed as the mean ± SD (*n* = 3).


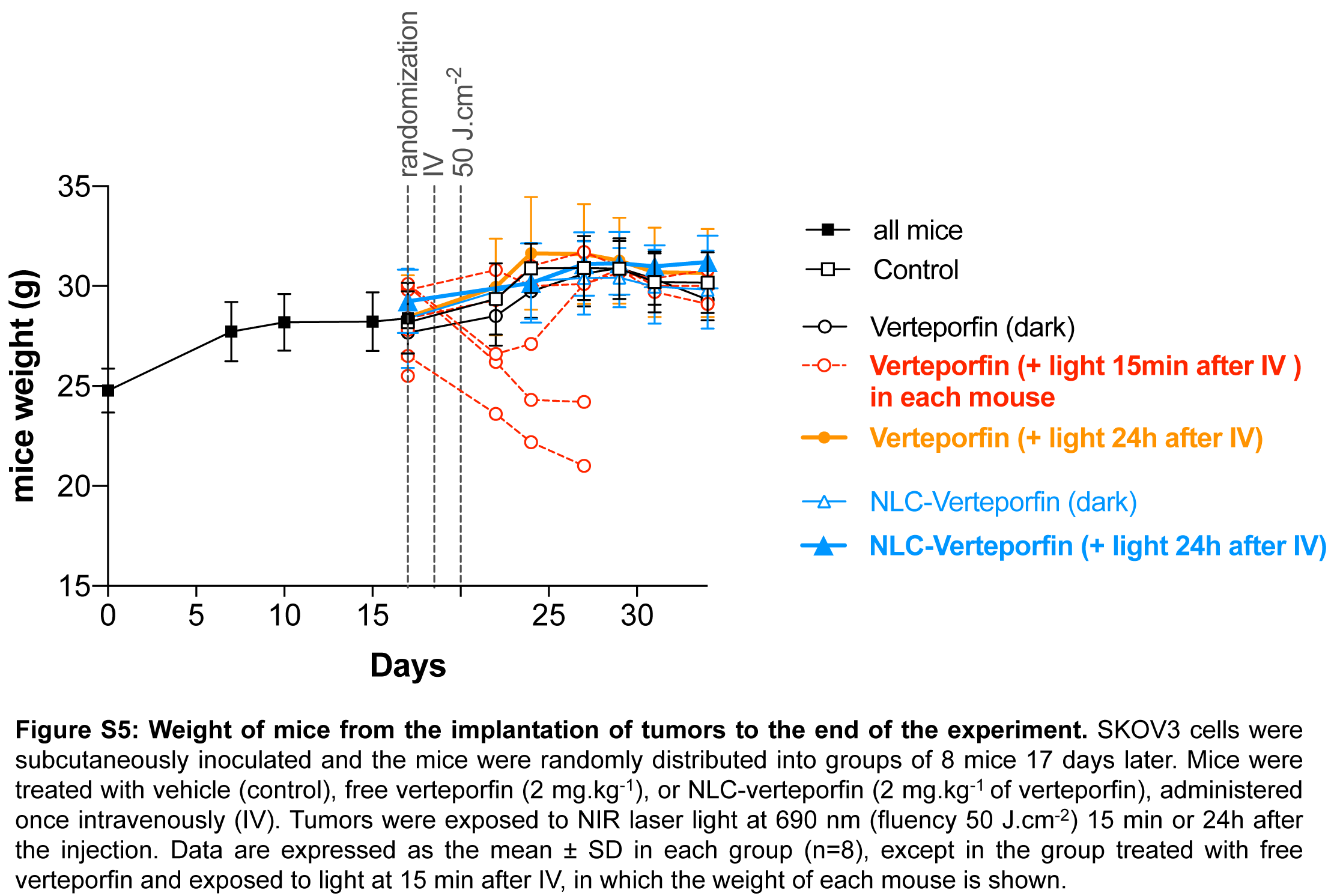


**Figure S5.** Weight of mice from the implantation of tumors to the end of the experiment. SKOV3 cells were subcutaneously inoculated and the mice were randomly distributed into groups of 8 mice 17 days later. Mice were treated with vehicle (control), free verteporfin (2 mg.kg^−1^), or NLC-verteporfin (2 mg.kg^−1^ of verteporfin), administered once intravenously (IV). Tumors were exposed to NIR laser light at 690 nm (fluency 50 J.cm^−2^) 15 min or 24 h after the injection. Data are expressed as the mean ± SD in each group (*n* = 8), except in the group treated with free verteporfin and exposed to light at 15 min after IV, in which the weight of each mouse is shown.


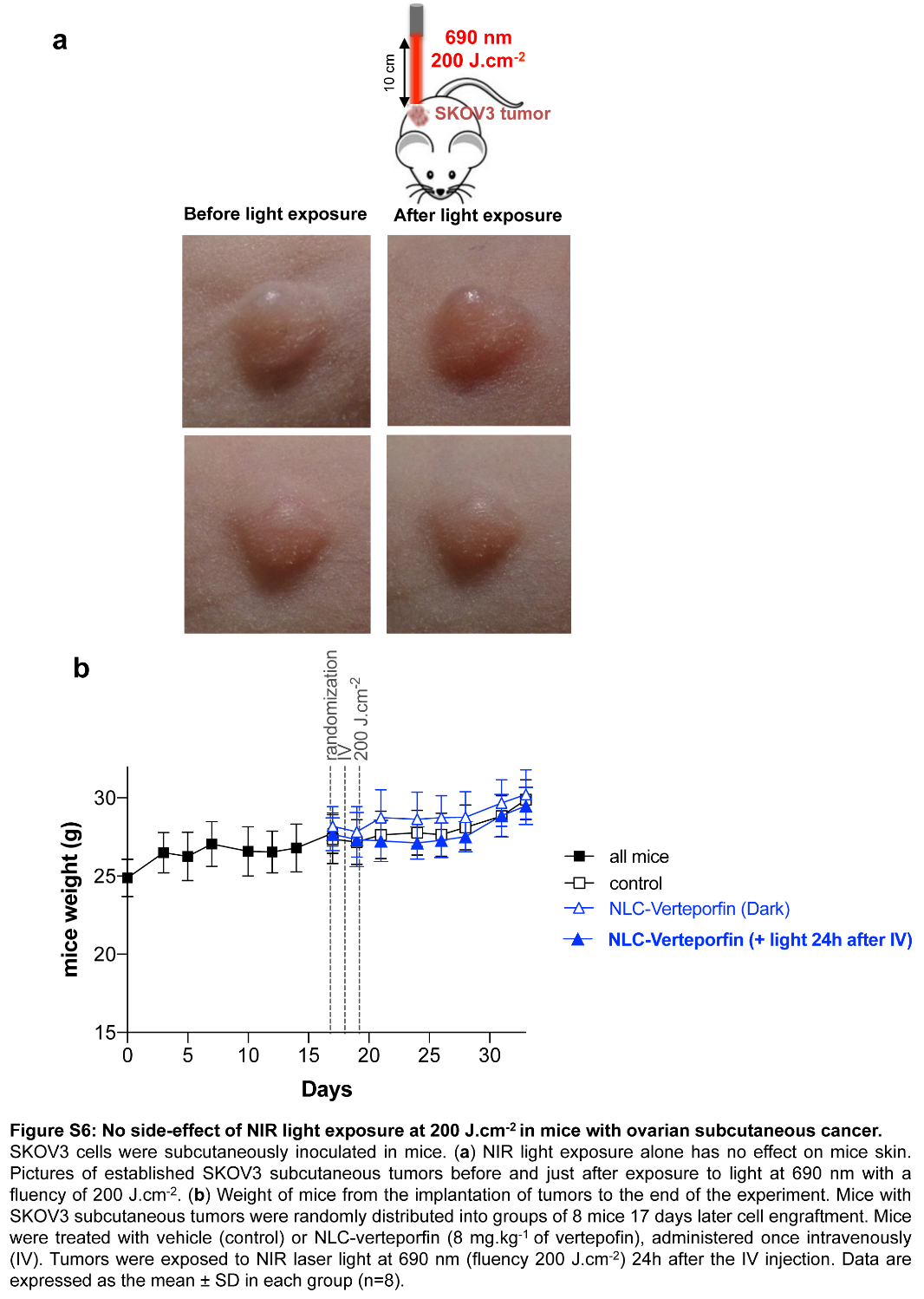


**Figure S6.** No side-effect of NIR light exposure at 200 J.cm^−2^ in mice with ovarian subcutaneous cancer. SKOV3 cells were subcutaneously inoculated in mice. (**a**) NIR light exposure alone has no effect on mice skin. Pictures of established SKOV3 subcutaneous tumors before and just after exposure to light at 690 nm with a fluency of 200 J.cm^−2^. (**b**) Weight of mice from the implantation of tumors to the end of the experiment. Mice with SKOV3 subcutaneous tumors were randomly distributed into groups of 8 mice 17 days later cell engraftment. Mice were treated with vehicle (control) or NLC-verteporfin (8 mg.kg^−1^ of verteporfin), administered once intravenously (IV). Tumors were exposed to NIR laser light at 690 nm (fluency 200 J.cm^−2^) 24 h after the IV injection. Data are expressed as the mean ± SD in each group (*n* = 8).


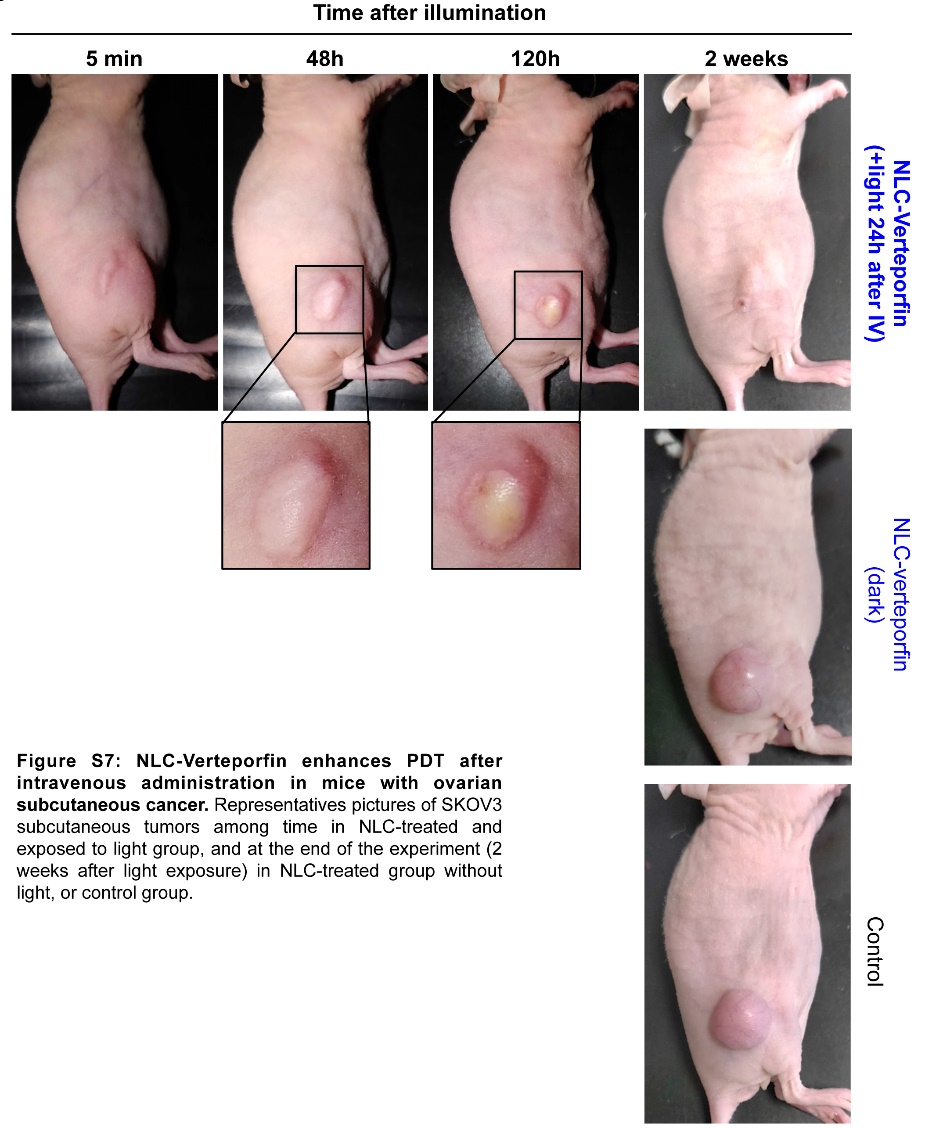


**Figure S7.** NLC-Verteporfin enhances PDT after intravenous administration in mice with ovarian subcutaneous cancer. Representatives pictures of SKOV3 subcutaneous tumors among time in NLC-treated and exposed to light group, and at the end of the experiment (2 weeks after light exposure) in NLC-treated group without light, or control group.


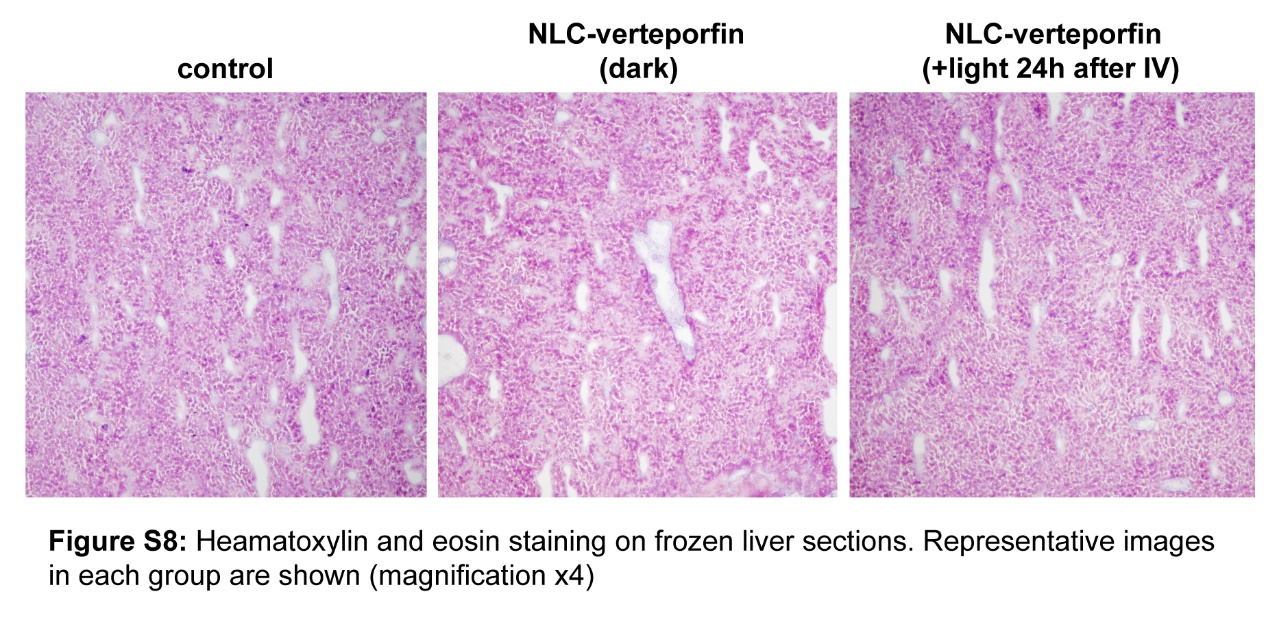


**Figure S8.** Heamatoxylin and eosin staining on frozen liver sections. Representative images in each group are shown (magnification ×4).


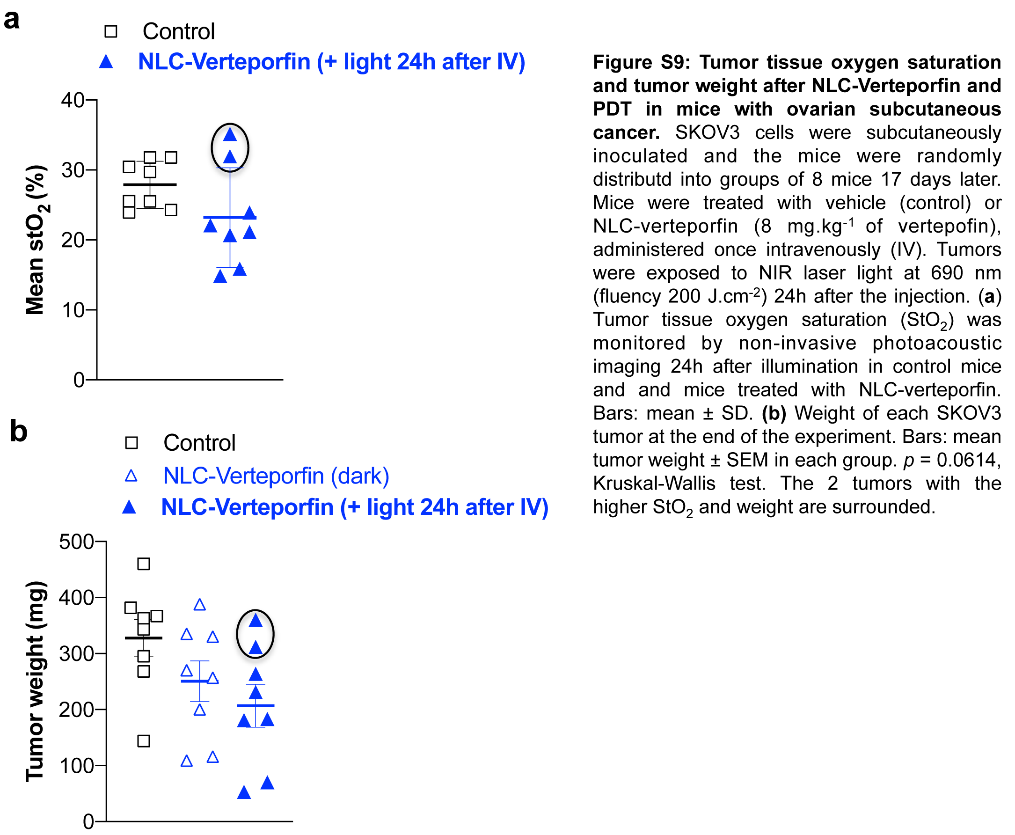


**Figure S9.** Tumor tissue oxygen saturation and tumor weight after NLC-Verteporfin and PDT in mice with ovarian subcutaneous cancer. SKOV3 cells were subcutaneously inoculated and the mice were randomly distributed into groups of 8 mice 17 days later. Mice were treated with vehicle (control) or NLC-verteporfin (8 mg.kg^−1^ of verteporfin), administered once intravenously (IV). Tumors were exposed to NIR laser light at 690 nm (fluency 200 J.cm^−2^) 24 h after the injection. (**a**) Tumor tissue oxygen saturation (StO_2_) was monitored by non-invasive photoacoustic imaging 24 h after illumination in control mice and mice treated with NLC-verteporfin. Bars: mean ± SD. (**b**) Weight of each SKOV3 tumor at the end of the experiment. Bars: mean tumor weight ± SEM in each group. *p* = 0.0614, Kruskal-Wallis test. The 2 tumors with the higher StO_2_ and weight are surrounded.
